# Temporal aggregation impacts on epidemiological simulations employing microcontact data

**DOI:** 10.1186/1472-6947-12-132

**Published:** 2012-11-15

**Authors:** Mohammad Hashemian, Weicheng Qian, Kevin G Stanley, Nathaniel D Osgood

**Affiliations:** 1 Department of Computer Science, University of Saskatchewan, Saskatoon, Saskatchewan, Canada; 2 Department of Community Health and Epidemiology, University of Saskatchewan, Saskatoon, Saskatchewan, Canada

**Keywords:** Infection transmission, Sensor-based data, Aggregation, Simulation model, Dynamic model, Contagion, Epidemiology, Surveillance

## Abstract

**Background:**

Microcontact datasets gathered automatically by electronic devices have the potential augment the study of the spread of contagious disease by providing detailed representations of the study population’s contact dynamics. However, the impact of data collection experimental design on the subsequent simulation studies has not been adequately addressed. In particular, the impact of study duration and contact dynamics data aggregation on the ultimate outcome of epidemiological models has not been studied in detail, leaving the potential for erroneous conclusions to be made based on simulation outcomes.

**Methods:**

We employ a previously published data set covering 36 participants for 92 days and a previously published agent-based H1N1 infection model to analyze the impact of contact dynamics representation on the simulated outcome of H1N1 transmission. We compared simulated attack rates resulting from the empirically recorded contact dynamics (ground truth), aggregated, typical day, and artificially generated synthetic networks.

**Results:**

No aggregation or sampling policy tested was able to reliably reproduce results from the ground-truth full dynamic network. For the population under study, typical day experimental designs – which extrapolate from data collected over a brief period – exhibited too high a variance to produce consistent results. Aggregated data representations systematically overestimated disease burden, and synthetic networks only reproduced the ground truth case when fitting errors systemically underestimated the total contact, compensating for the systemic overestimation from aggregation.

**Conclusions:**

The interdepedendencies of contact dynamics and disease transmission require that detailed contact dynamics data be employed to secure high fidelity in simulation outcomes of disease burden in at least some populations. This finding serves as motivation for larger, longer and more socially diverse contact dynamics tracing experiments and as a caution to researchers employing calibrated aggregate synthetic representations of contact dynamics in simulation, as the calibration may underestimate disease parameters to compensate for the overestimation of disease burden imposed by the aggregate contact network representation.

## Background

Computational models of contagion can provide insight and foresight into the behavior of particular pathogens for specific populations. The representation of contacts between population members has a critical impact on simulation outcomes [1,2]. It has long been recognized that models that assume random mixing between aggregate population categories can alter the dynamics and results of the simulation of contagion spread [3], and may inhibit insight into intervention trade-offs [4]. Reflecting such findings, the past decade has witnessed the growing use of agent-based transmission models, which can better capture the impact of individual-level contact patterns on both individual risk and population-wide transmission dynamics. Such models have frequently represented the inter-agent contact patterns as static graphs with edge weights corresponding to threat of infection drawn from uniform, power-law [5], or other [5,6] distributions.

More recently, researchers have used microelectronic devices such as motes [7-9], RFID tags [10], cell phones [11-14], or custom-built wireless technologies [15] to capture contact distance, duration and location, as well as other parameters, with significantly more accuracy than previous diarying [16,17] or retrospective self-report [18,19] methods, and substantially greater accuracy than cellular [20,21] or WiFi [22] location estimates whose modeling use entails assuming random mixing patterns between individuals in the same or nearby geographic regions. Data collected from such dynamic contact networks has been used to simulate the spread of a pathogen through the recorded network, using both the raw sample data [15,23,24], and aggregated versions where infection probability is drawn from an empirical rather than functional distribution [9]. While electronically supported micro-contact data collection offers significant spatial-temporal resolution and compliance advantages over traditional techniques, the relatively high cost and logistical effort involved in a deployment of telemetry systems and their limited on-board energy capacity imposes design tensions between study duration, sampling rate, and participant count. While study design has been shaped by clear cost-economic considerations favoring shorter deployments with larger populations [9] or longer deployments with smaller populations [8,23], it has been been conducted absent a clear understanding as to how study design or post-study data aggregation affect transmission model accuracy. Reflecting the power hungry character of the data collection devices, studies have frequently favoured larger, high sampling-rate designs of just one [9], two [10,25] or a handful of days [26] in duration. While some researchers have argued that such short sampling periods capture one or more typical day(s) [9], there remains a dearth of formal evidence for the representative character of short sampling periods, and the effects of limited sampling periods on the quality of subsequent simulation outcomes have not been explored.

Researchers have employed diverse strategies for using micro-contact data in transmission models. In some cases [25], researchers have sought to use traditional static representations, with weights on the link connecting two individuals being proportional to the cumulative contact duration observed between those individuals over the study period. Other studies [9,23,24,27] have employed strategies in which dynamic contact patterns collected over an interval of time are used directly as the contact pattern for that the same time horizon. In initial examinations of the effects of aggregation of microcontact data on simulation outcome, the authors of [28] noted the effects of aggregation on contagion using two sets of empirical data, and the authors of [25] explored the impact of changes in model parameter values and network aggregation measures – including alternate use of both weighted and unweighted static representations – to simulation outcomes. Because the dataset in [28] was collected over a relatively short (2 day) study duration, the researchers replicated the recorded data and variants over longer simulation horizons (60 or 100 days) to ensure sufficient duration to capture the dynamics of the simulated outbreak, and found that application of unweighted homogeneous aggregation schemes led to significant overestimation of infection spread. The study further examined three strategies for extending the time frame, with all three approaches positing that the observed contact recorded over the brief study period are representative of a longer period of time. The researchers found notable epidemic-phase-specific differences between the typical day [9] variants, reflecting whether contacts occur repeatedly with the same set of individuals or different individuals. Most importantly, simulated infection transmission on a duration-weighted aggregated static graph yielded results close to those from a fully disaggregated but replicated dynamic network. However, because of the short study duration, the researchers were unable to evaluate the fidelity of any of the three approaches to simulated infection transmission over a disaggregated dynamic network for the entire simulation time horizon. While this contribution yielded important insights into the effects of aggregation and replication strategies on simulation outcomes, the researchers emphasized the importance of analyzing data from longer-duration studies, to validate the fidelity of the proposed strategies for study data extrapolation.

To help address and expand upon the questions raised in [25] regarding the impact of temporal aggregation and extrapolation, this paper analyzes such effects for the Flunet dataset [8] in the context of an influenza-like illness (ILI). The Flunet dataset is characterized by a two minute-level temporal contact resolution, and a smaller population (N=36) observed over a much longer time period (T=92 days) than other studies [9,10]. Taking advantage of the longer time duration of this study, we examine agent-based simulation using a previously contributed ILI model [29], and both previously contributed [8,23,24] and novel dynamic network structures, and previously used but unevaluated strategies for extending the study timeframe. We report three important findings:

Even the weighted “typical-day” techniques advanced in [9,25] are fragile because the variance in contact patterns between days can lead to highly variable simulation outcomes;

Confirming with an extended temporal horizon and extending findings of [25], aggregated network representations tend to overestimate the disease burden in the population;

While static synthetic networks with contact distributions can produce results consistent with the full dynamic network, this apparent agreement can be due to an accumulation of counter-balancing inaccuracies leading to a deceptively correct outcome.

Overall, our findings suggest that longitudinal data collected over a prolonged period is required to accurately reconstruct dynamic or static contact networks for the purpose of simulating pathogen spread for at least some subpopulations, and that simulations based on summary networks tend to systematically overestimate the risk of infection. These findings are important because they suggest that traditionally calibrated disease models may contain biased disease parameter estimates to compensate for undetected inaccuracies in the underlying network and mixing models. These biases in disease parameter estimates might then lead to erroneous conclusions about the relative impact of interventions or the rate of disease propagation. Because such inaccuracies may not cancel in the same fashion when investigating intervention effects, additional caution should be employed when using such aggregation and generalization schemes. Unfortunately, while typical day [9,25] contact data acquisition techniques ease study design, they do not appear to be an adequate solution, as they can yield even more erroneous disease burden estimates than traditional aggregate models. This leads us to finally conclude that larger-scale longer-term longitudinal studies may be required to generate sufficiently accurate descriptions of the contact dynamics in many populations. Such studies are urgently needed to help identify the balance between study size and length required to secure reliable insight into intervention tradeoffs.

## Methods

Given the H1N1 strain emergence and in anticipation of the significance of the 2009–2010 influenza season, the co-authors launched a previously-described [24] pilot study in the midwestern Canadian city of Saskatoon to electronically collect contact patterns between 36 participants, in addition to their influenza-related health status information. The study was conducted between November 9th, 2009 and February 9th, 2010 – a time period coinciding with the second rise of reported H1N1 cases in the province of Saskatchewan [30]. Each participant was requested to carry a proximity sensor whenever awake during the study period, and to respond to a sequence of weekly health surveys via a web browser. Participants filled out an informed consent form prior to joining the study, as required by the university research ethics board.

To study the impact of network representation on simulation outcomes, an H1N1 infection transmission model [24] was created and parameterized with empirical characteristics of the H1N1 pathogen [31]. The model simulated the spread of infection between agents through their daily interactions. Agents’ daily connectivity – either from empirically collected or aggregate approximations – were imported into the model and defined the potential for infection between discordant agents. Overall, 616 different scenarios – each positing a different connectivity network derived from the data – were simulated, with each such scenario being run for 10,000 realizations by replaying a sequence of the 265,000 thirty-second time slots in the study period. Based on the 100,000 run ensembles with the same model and dynamic network reported in [24] we concluded that 10,000 runs were sufficient, as the maximum depth of infection in [24] was reached 10 times, implying that division by 10 would have a high probability of capturing even the most extreme infection events, while maintaining the probability distribution at significantly reduced computational cost. Because each run could be conducted relatively swiftly (typical run-times for an ensemble were 30 s) having a large number of runs was not computationally prohibitive given the resources available to us.

The underlying disease parameters and distributions were held constant across scenarios. Within a given scenario, stochastics associated with exogenous and endogenous infection transmission and duration of different phases in the natural history of infection induced variability in simulation output.

### Data collection

The Flunet experiment covered 57 weekdays and 33 weekends/holidays (including Christmas break). Participants were asked to carry a small wireless sensor (or “mote”) capable of short-range wireless communication [8]. When two motes were in close proximity, they would record a contact with a minimum resolution of 30 seconds. Each contact record represented a contact session between two motes, which included the start and end time of a contact, and the distance between the adjacent motes binned by the received signal strength indicator (RSSI, a measure of the wireless signal strength) into close (< 5 m), medium (5–15 m) and far (> 15 m) bins [8]. Contacts longer than 7 hours (0.03% of total reported contacts) were removed, as we assumed that most contact of this duration was due to sensors abandoned near each other making the contact duration distribution broadly consistent with other long-term datasets [8,23,32]. Participants were asked to fill out a sequence of weekly health surveys, which included symptoms and diagnoses of ILI, reported date of H1N1 vaccination, and self-reported contact patterns. Demographic data was collected in a single survey at the conclusion of the study.

### Transmission model

As the data collection occurred during the H1N1 outbreak, we used an agent based H1N1 SEIR transmission model to simulate the infection dynamics. This section includes a short description of the model; interested readers are referred to a detailed specification in [24]. The simulation model classified each individual in the sample population into one of seven states: Susceptible, Latent, Asymptomatic Infectious, Symptomatic Infectious, Symptomatic Non-Infectious, and Recovered. All the agents in the model started in the Susceptible state, consistent with limited pre-existing population-level immunity to H1N1. A susceptible individual could contract the infection either from exogenous or endogenous sources. Exogenous sources are defined as the population outside the study who were in contact with Flunet participants and could transmit the infection to the monitored individuals, while endogenous sources are other Flunet participants in an infectious state within the simulation. Both endogenous and exogenous infection forces of infection were calculated using data from the 2009 outbreak [30], a prominent H1N1 model [31], and collected contact data [24].

**Figure 1:**
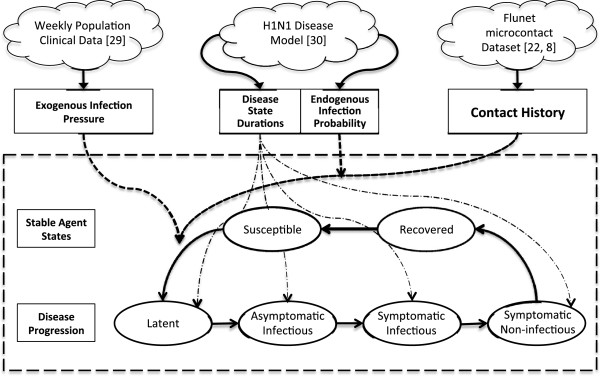
**Simulation structure and flow.** This figure demonstrates the agent state transition process (oval nodes) and model parameter sources (clouds) as a flow process. Parameters inform the probability of state transition changes.

A susceptible agent contracting the infection from either exogenous or endogenous sources transitions to the Latent state. When an individual enters the Latent period, the model computed the duration for each of the subsequent four stages of illness (Figure 1). In determining these durations, we sought to reproduce the observed variability in H1N1 progression by drawing the duration of incubation and duration of symptoms from two log-normal distributions [24]. The duration for other stages were calculated using these two values.

Each infected agent experienced the four illness states sequentially with the passage of time. A person in the Asymptomatic Infectious or Symptomatic Infectious state was considered infective. At each time the infective person triggered potentially transmitting events (e.g. coughs or sneezes) with a specified likelihood. A potentially transmitting event had a given probability of transmission to each susceptible in contact. The simulation model did not consider H1N1 mortality, self-quarantine, antiviral administration, or hospitalization outcomes. While the H1N1 model employed has been previously used the capture the impact of vaccination [24], vaccination was not considered in this study.

### Connectivity patterns

This work seeks to analyze and quantify the impact of contact pattern representation on transmission outcomes in agent-based simulation models. Because we had recourse to data offering considerably greater temporal span than most other past contributions in this area, we could more readily examine the impact of varying levels of temporal aggregation on model outcomes. To do so, we looked at three different experimental manipulations, each associated with additional parameter variations focused on a particular type of network representation. A combination of manipulation and parameter variation is termed a scenario.

The first baseline manipulation focused on two reference scenarios, each representing different extremes in the aggregation spectrum. The first employed the Flunet empirical data directly, as it captures the contact patterns between agents with the greatest fidelity. In the dynamic baseline graph, edges have weights of 1 or 0 – that is, they correspond to the existence or absence of a connection during a given timeslot. The dynamic contact network can be visualized as a series of network where the nodes represent participants and each connections represents a pair of participants that were proximate to each other during a time step. This construct is often called a dynamic graph – drawing from the field of Graph Theory – where the participants are nodes, and the connections are the edges. This graph can either be realized in practice as a sparse dynamic graph with edges appearing and disappearing, or a time series of symmetric matrices with binary constituents, with 0 indicating the absence and 1 indicating the presence of an undirected edge.

The second baseline manipulation scenario collapsed the dynamic Flunet graph down to a single static graph. In the static graph scenario, edge weights are replaced by the time averaged contact density between a specific pair of participants over the entire study. This is trivially constructed as the sum of all the dynamic symmetric matrices divided by the number of timesteps. This aggregation approach captures an extreme form of aggregation, in which no changes are made in contact graph structure over time. If contact dynamics had no significant impact on the results, then simulations using this graph should echo the fully dynamic case.

The second manipulation examined typical day approaches. As a method of temporally extrapolating graphs collected over shorter time horizons, both [9] and [15] employed a typical day [9] hypothesis, positing that the day(s) captured in the experiment were generally representative of longer-term contact patterns and could be duplicated through time to extend the dynamic graph until the simulated outbreak dissipated or reached a quasi-static endemic equilibrium. Once an interval was selected, the day-by-day duplication of that interval could be accomplished in one of two ways [9,25]. In the first approach, the collected contacts are aggregated over that interval – in a manner similar to how the static graph is aggregated over the course of the study – with the aggregated contact network then being applied across the entire simulation time horizon. Another appoach simply replicates the fully dynamic graph for that interval throughout the study period. To evaluate the impact of the typical day hypothesis, the second manipulation consisted of a series of simulations with duplicated days using both the fully dynamic and aggregated typical day scenarios.

Reflecting the fact that many researchers lack recourse to high-fidelity empirical contact data for model integration, the third manipulation focused on fitted synthetic networks. Past contributions have generated random contact networks using small-world, scale free or other network topologies, often with contact probabilities represented as edge weights randomly drawn from other independent distributions. To analyze the impact of such an aggregate network representation on the spread of infection across the simulated population, the dynamic Flunet graph was reduced to distributions for edge weights and node degree and a set of new small-world contact graphs based on these distributions were created. To determine the impact of model fit on simulation results, several edge weight distributions were employed that provided increasingly accurate fit, at the cost of decreasing theoretical rigour.

**Figure 2:**
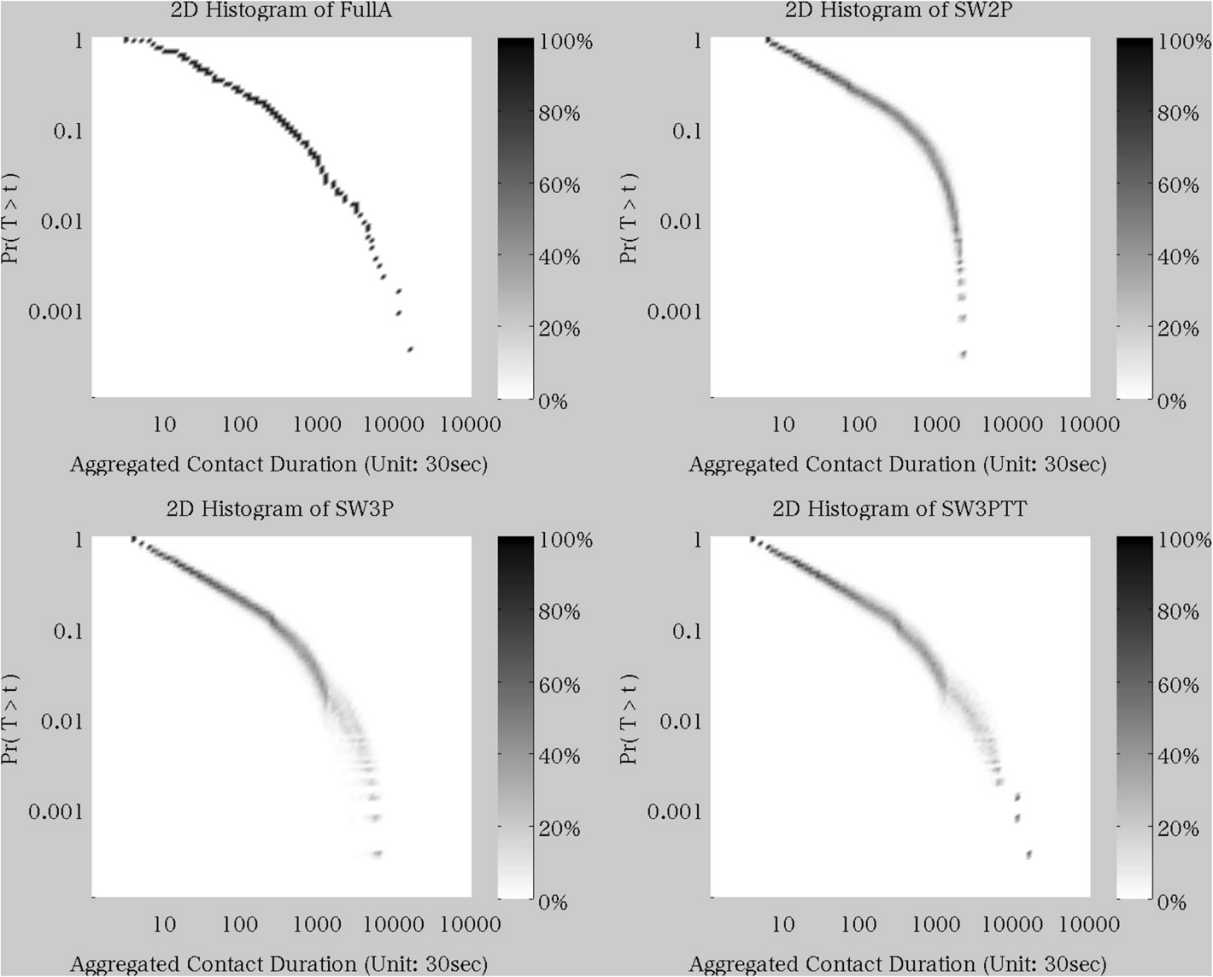
**Histogram for Distribution of Generated Aggregated Contact Duration.** This figure provides a heat map to demonstrating the distribution of generated aggregated contact durations used in the simulation. Each sub-graph represents the CCDF of contact duration for: **a**) the empirical distribution used in the FullA case; **b**) the two part power-law exponential distribution commonly used in practice; **c**) the three part power law-exponential-exponential distribution which provides a better fit to our data; and **d**) the best fit power law-exponential-exponential with outliers included as single empirical data points. The shade of the point represents the frequency with which a network contained exactly that contact duration-probability pair. Some slight fanning of the distribution in **b**, **c** and **d** at higher contact durations indicates that our network construction algorithm had good but not perfect reconstruction of contact durations when compared to the empirical baseline.

Unweighted small-world connectivity graphs with 36 nodes were generated using the Watts and Strogatz model [33] in R [34,35]. Average path length and cluster coefficient were used as measures of similarity with respect to the aggregate empirical Flunet graph. Five hundred possible parameterizations of small-world networks based on different connectivity ranges and rewiring probabilities were each generated with 10,000 realizations. For each such realization, the average of each of the similarity measures were calculated. The connectivity range and rewiring probability parameterization that yielded the highest average similarity measure was selected as the base for unweighted graph generation. The parameters were then used to generate final unweighted small-world networks. Figure 2 shows the resulting distributions of networks selected using this process.

Weights were assigned to the edges of the unweighted small-world network by drawing the value for each edge from a distribution. For each of the scenarios within the synthetic network manipulation, we examined the effects of using three different fitted distributions, as well as drawing from the normalized empirical histogram of pairwise Flunet aggregated contact durations (ACDs).

Fitting of distributions was performed using linear regression in MATLAB. For linear regression to perform properly, data underwent log-log (power law fitting) or log-linear (exponential fitting) transformation prior to performing the piecewise fit. For fitted distributions, the fitted curves were required to achieve a R^2^ value exceeding 99%. Piecewise breakpoints were selected by iteratively changing the breakpoints, performing the regression, and manually selecting the point at which error began to increase sharply, but which still maintained a minimum R^2^ of 99% value for all the piecewise components.

**Figure 3:**
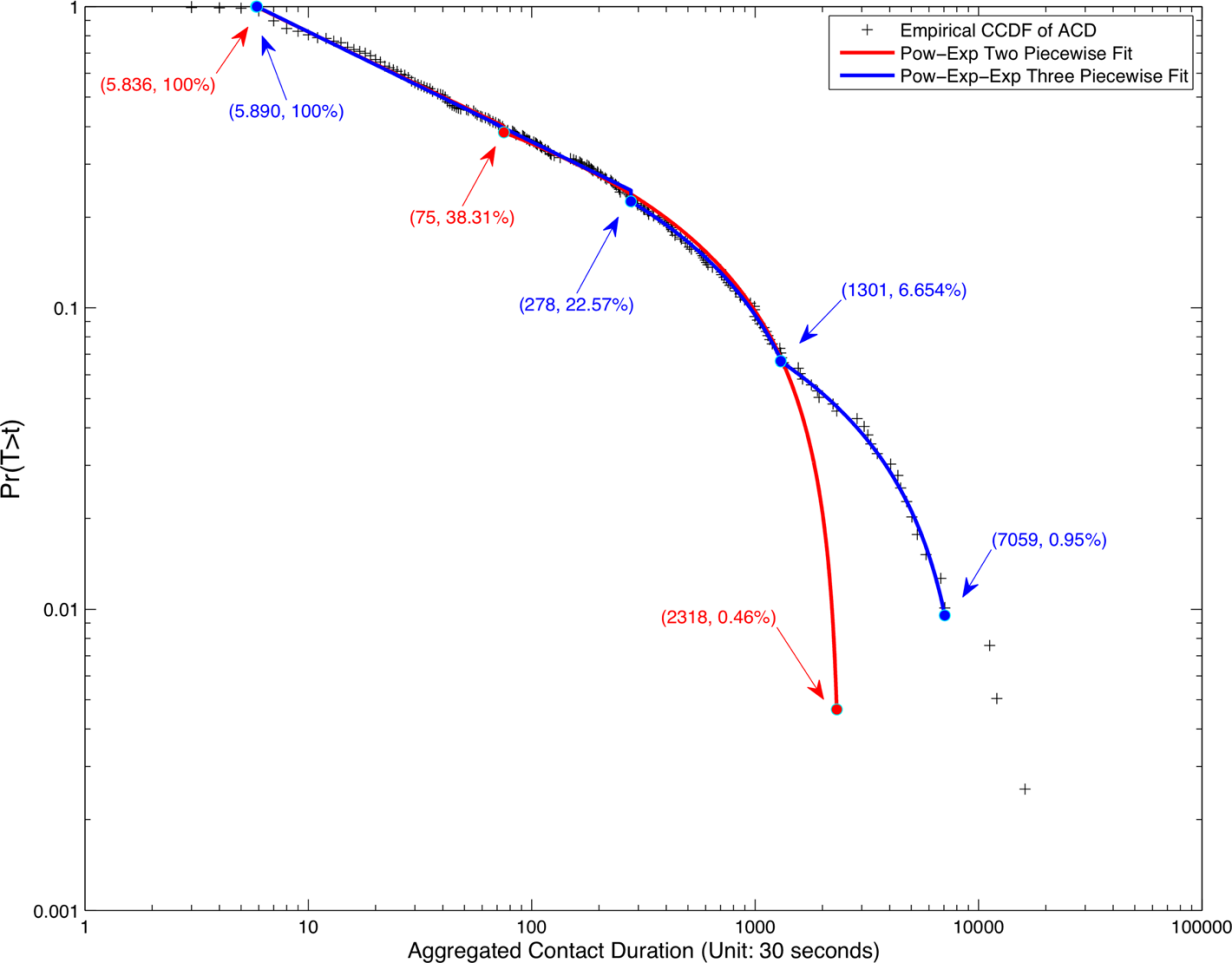
**Curve fitting methods of Aggregated Contact Duration.** This figure shows the four piece-wise fitting approaches for aggregated contact duration over a CCDF plot of the empirical data. Points represented by a (+) are indicative of the empirical probability of a contact duration. The solid red line corresponds to the two-piece power law-exponential distribution fit. The solid green line corresponds to the three-piece power law-exponential-exponential distribution fit. Text in blue or red corresponds to the break points for each fit section. Outliers (at the head and tail) not included in either fit are included in the SW3PTT case.

Figure 3 shows one of the curve fits implicitly and three explicitly. The red and blue lines correspond to the two and three piece fits for the data. The data points themselves form a discrete empirical distribution, and the three piece plus the additional three points with the highest aggregated contact duration form a combined functional-discrete distribution, which can be mathematically considered a 6 part piecewise function, where the discrete outliers are described as Dirac delta functions.

Infectious events were generated according to a Poisson process as described in [24]. When such event occurred in the FullD and DayD cases, the state of the contact graph was queried, and connected agents were infected based on the probability of infection for the pathogen. For aggregate representations a joint probability of the edge weight from the static graph and infection probability was used to determine infection probability. This is mathematically equivalent to randomly sampling the contact records from either the DayD or FullD contact records for the DayA and FullA cases.

### Detailed scenario description

The three primary experimental manipulations (baseline, typical day and synthetic network representations) are each represented by a set of scenarios describing the method for generating the contact graphs. Each scenario in turn is composed of a set of cases; for example, there are 57 possible typical day pairs to investigate using our methodology; by contrast, each baseline scenario is associated with just a single case. Each experimental case was simulated for 10,000 realizations, where a single realization is an agent-based simulation of the entire 92 day study period, yielding a total of 6.16 million realizations across all scenarios. Here we describe each scenario in detail.

1. **Full-Detailed Network (FullD):** FullD is the first scenario in the baseline experimental manipulations. The connectivity pattern in this case uses the complete contact information of participants throughout the study as a dynamic graph, preserving the chronological order of contacts. For each participant, his/her observed contacts with other individuals in the study at each of 265,000 thirty-second time-slots across the 3 months of the Flunet study were imported into the model to represent the connectivity pattern of the corresponding agent. In each realization, the model stepped through all timeslots sequentially, simulating infection transmission using the corresonding inter-agent connectivity graph.

2. **Full-Aggregated Network (FullA):** FullA provides an upper bound on the impact of temporal aggregation. The connectivity pattern of this scenario is similar to the FullD scenario, but the contacts are aggregated over time. To generate the connectivity pattern, the study-wide per-timeslot contact likelihood between any two given agents was calculated based on the Flunet database, and imported into the model. Assuming the probability of two given nodes contacting each other across the entire 92 day study period is *p*, the connectivity between those two agents in a given timestep model was drawn from a Bernoulli distribution with success rate of *p*. Before starting the simulation, 265,000 samples were drawn from the distribution for each pair of nodes, representing the simulation connectivity patterns between those nodes during each successive timestep. Note that, in this scenario, contact patterns are regenerated at the beginning of each realization, and while the contact likelihood between pairs of agents are held invariant between realizations, the network dynamics and chronological order of the contacts are not preserved.

3. **Day-Detailed Network (DayD):** The Flunet study covered 57 weekdays and 34 weekends/holidays. The DayD scenario – the first scenario in the typical day manipulation – abstracts this as 57 cases, each related to a unique pair of weekday-weekend/holiday. The first 34 weekdays paired with each of the first 34 weekend/holidays, and the remainder of the weekdays are paired by repeating the first 23 weekend/holidays. Subsequently, each pair was used to generate the connectivity pattern between agents by replicating the weekday of the pair 57 times (for the non-holiday weekdays during the study period) and replicating weekend/holiday of the pair 34 times (during the weekends and holidays during the study period). Therefore, in each case of this scenario, the ordered contacts of a particular weekday-weekend pair were replicated to cover all remaining days of the simulation as well.

4. **Day-Aggregated Network (DayA):** This scenario – the second scenario in the typical day experimental manipulation – consists of a hybridization between DayD and FullA. Like DayD, it also consists of 57 cases, each based on one of the 57 weekday-weekend pairs. Similar to FullA, the contact network used is aggregated over time (here, over a day) and regenerated by each realization prior to simulation. For each of the 57 weekday-weekend pairs, we derived the weekday-specific per-timestep contact probability between any two given nodes based on the specific contact patterns seen in the weekday from that pair. A weekend-specific per-timestep contact probability was analogously derived for each pair of nodes. For each pair of participants, and each timeslot of each (non-holiday) weekday throughout the study period, samples were drawn from a Bernoulli distribution with the specified weekday-specific contact probability for that pair. Contacts in weekend timeslots were similarly defined.

5. **Small-World Network with Power Law-Exponential Fitted ACD (SW2P):** In the first scenario in the synthetic network experimental manipulation, the selected connectivity range and rewiring probability (described in the previous section) were used to construct 100 unweighted small-world networks. To determine the weights for edges, a distribution was generated using a two-piece Power Law-Exponential distribution fitted to the Flunet ACD curve based on [36], where 4 data points from the head and 18 data points from tail of the empirical distribution were cut to improve the fit. The connectivity of network *i* was determined by drawing the weight associated with each edge in the unweighted *i*^*th*^ small-world network from the 2 piece distribution.

6. **Small-World Network with Power Law-Exponential-Exponential (SW3P):** To construct connectivity patterns in the second scenario of the synthetic network manipulation an approach similar to SW2P was followed, but a 3-piece Power Law-Exponential-Exponential distribution was used to fit the Flunet ACD empirical distribution. Here, 3 data points from the head and 4 data points from the tail of the Flunet distribution were removed to improve the fit. As with SW2P, 100 connectivity networks were created.

7. **Small-World Network with Power Law-Exponential-Exponential True Tail Replaced (SW3PTT):** In the curve-fitting for SW3P – the third scenario in the synthetic network manipulation – we cut 4 points from the tail to better fit the distributions. Although the eliminated tail portion is approximately 1% of all data points in the Flunet empirical distribution, their contact duration was substantially larger than that of other data points, resulting in a greater importance to the network-wide transmission of infection [24]. Similar to SW3P, this scenario also generates 100 connectivity networks by drawing from a 3-piece fitted distribution, but randomly selects 4 weights within each such network and replaces them with the 4 empirical values to preserve the important contacts in the tail.

8. **Small-World Network with Empirical ACD (SWE):** The fourth and final scenario in the synthetic network manipulation uses a specified connectivity range and rewiring probability to generate 100 small-world networks, where the weights for the edges in each network are drawn from the empirical Flunet ACD distribution.

## Results

### Flunet dataset characteristics

**Figure 4:**
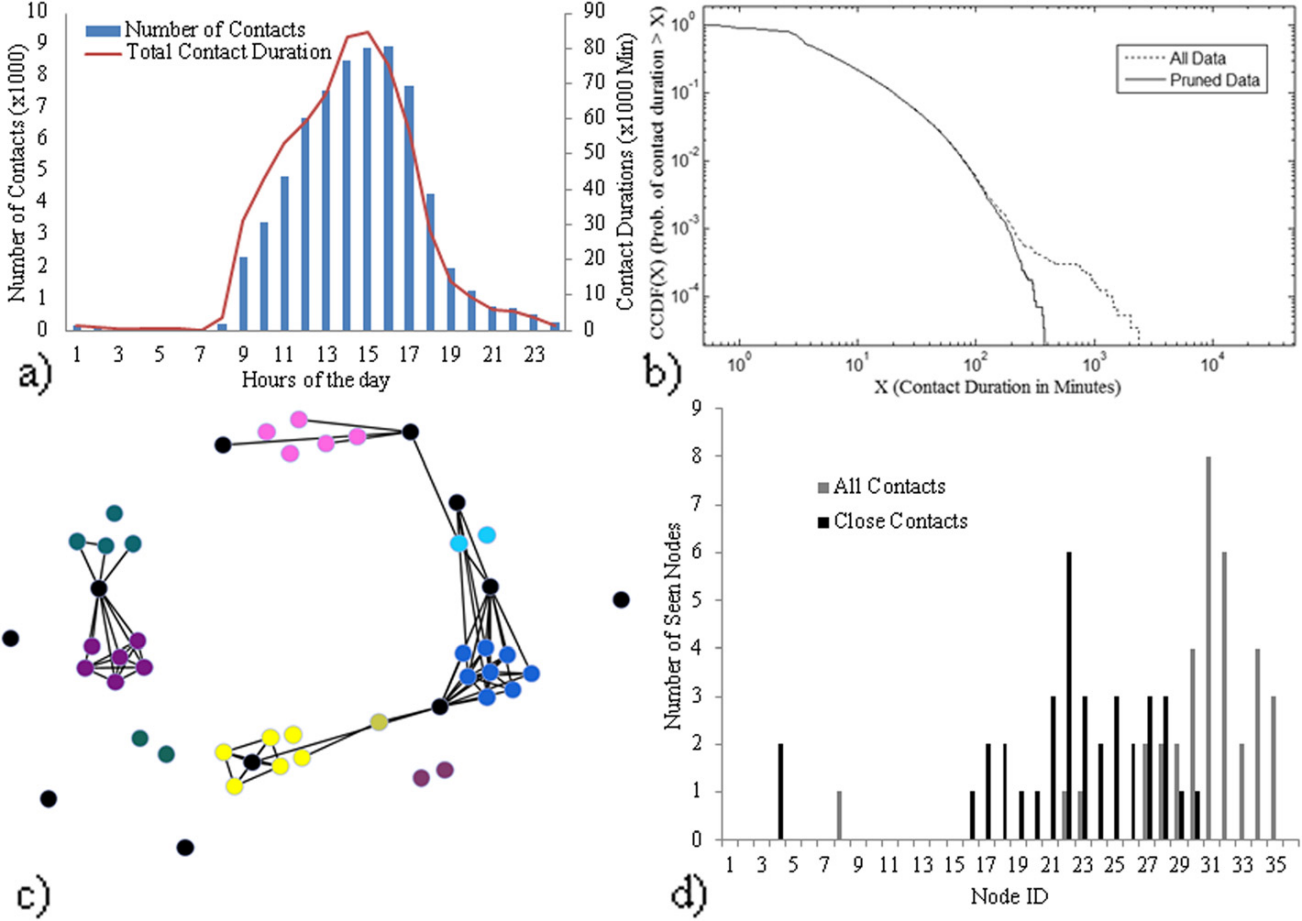
**Flunet Findings.** Flunet Findings **a**) Contact histogram by hour of day, **b**) CCDF of contact duration, **c**) Connectivity graph with threshold of 18 minutes per day average contact. Black nodes represent stationary nodes associated with a location, and are included in this graph for illustrative purposes only. **d**) Network span for close and all contacts.

A preliminary analysis of the dataset is provided in [8], and a similar analysis found in [24]. The majority of the description of the Flunet dataset in this subsection, including Figure 4 in its entirety, is a direct reproduction from [24], included here to increase the readability of the paper. Additional discussion germain to the analysis of the impact of network representation on infection rate has been added. The remaining subsections of Results contain new figures and analysis based on the simulations described in the Methods section.

Figure 4.a shows that contacts are tightly clustered throughout the workday, with staff arriving in the morning and graduate students trickling in throughout the day. Sporadic contacts are recorded throughout the evening and night. This figure also illustrates that the contact data contains primarily workplace relationships. While the contact densities in 2.a hold in general for those times when contact occurred – normally weekdays – they do not adequately capture the patterns on the weekend or during holidays (particularly the week between Christmas and New Year’s Day) which are characterized by more sporadic or sparse connections. However, it is worth noting that these gaps in connectivity relate to the underlying contact patterns of the sample, which are characterized by primarily professional relationships. While we cannot claim that a given participant will have a diminished chance of infection from over a weekend or holiday, we can claim with a great deal of certainty that they will have a diminished chance of becoming infected by a co-worker during those time periods. Figure 4.b shows the complementary cumulative distribution function (CCDF) of contact duration. In addition to the CCDF for all the collected data (solid line), we removed contacts with durations exceeding 7 hours (0.03% of total reported contacts) from the raw dataset (dashed line) because we assumed contact of this duration was due to sensors abandoned near each other. Removing this section of data yields considerable differences in the distribution’s tail. The CCDF is broadly consistent with other long-term datasets of this nature [16]. The heterogeneity of the contact distribution is important for our hypotheses and assumption – that contact dynamics have significant impacts on infection rate as initially noted by [6] – which in turn drove the simulation design. In Figure 4b, contact duration spans more than two orders of magnitude. If we posit that an infectious individual gives rise to contagious events (e.g., sneeze or cough) with some stochastic arrival probability independent of the contact duration, a susceptible is likely to experience more contagious events in a prolonged contact than in a shorter one, a property assumed in some other modeling studies [17].

To visually highlight the impact of cliques and place on the dataset, Figure 4c plots the relationship between contacts which existed for an average of 18 min/day. This threshold was to represent a plausible amount of time per day that a regular contact might have occurred over the course of the study, bearing in mind that weekends and holidays are included in the denominator. Black nodes represent stationary nodes associated with a location, and are included in this graph for illustrative purposes only. As is apparent in the graph, nodes that are generally collocated have a high degree of contact with each other. Nodes that are not collocated have much lower connectivity, with the exception of a few bridging individuals.

Given the importance of network structure, we consider the span of the network in Figure 4.d, which is closely related to degree centrality [24]. This graph is shown for two scenarios: a scenario where only close proximity constitutes a contact, and a scenario where any detectable presence qualifies. When limiting the analysis to close contacts, the histogram is both more peaked and has a lower mean than when considering all the possible contacts. The modes are 22 and 31, respectively, implying that many participants saw most of the other participants at least once. However, because it is not saturated at the maximum (as would be the case if all participants saw all other participants) it is logical to hypothesize that some partially isolated cliques exist, and that the close contact network is more strongly cliqued.

### Contact pattern representations effects

We sought to study how the representation of dynamic contact networks over time impacted simulated transmission of an influenza-like illness. In particular, we were interested in determining whether the choice of a sample day as a typical day had an impact on reliability of results, whether significant differences in outcome were observed when collapsing empirical contact durations into distributions, and whether representation of dynamic – rather than aggregate – contact networks significantly affected simulated outcomes.

**Figure 5:**
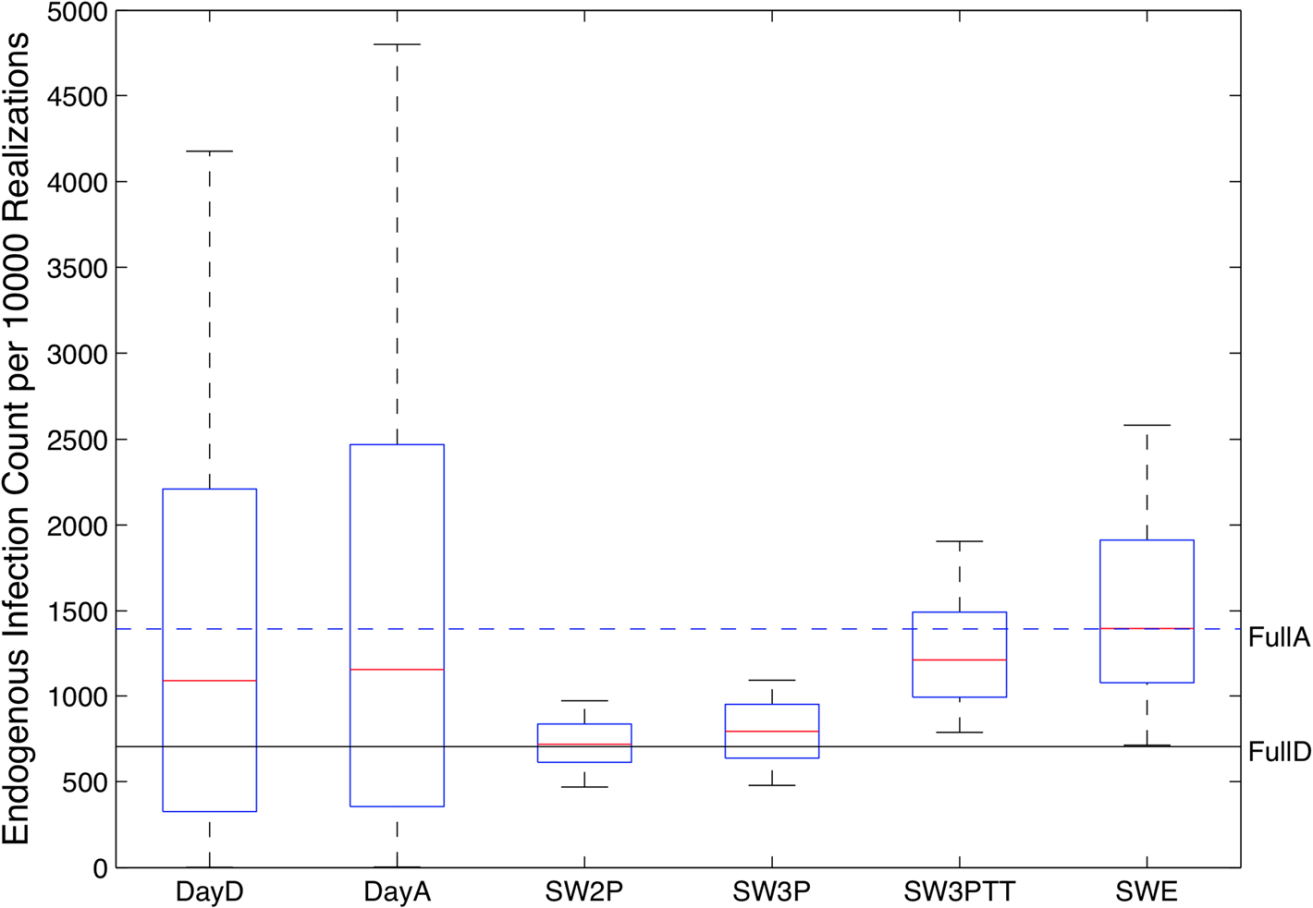
**Boxplot of Infected Count.** The number of endogenous infections of each realization summed by case are displayed as boxplots, and the FullA and FullD scenarios are displayed as single-case lines. The two day cases are represented on the left, have means between FullA and FullD scenarios and substantial variance. The synthetically created networks move increasing fidelity with the FullA case from SW2P (the weakest contact duration fit) to SWE, based on the empirical distribution of contact durations.

To compare overall trends in the findings, a simulation outcome for each scenario described in the previous section was graphed as a box plot in Figure 5. The y axis shows the total number of endogenous infections observed in the population, summed over all 10,000 realizations for one case. FullD and FullA scenarios are represented as straight lines across the graph, as they were each composed of a single case, providing useful reference bounds for the other ensembles. The other 5 scenarios are plotted as boxplots over 57 data points (DayD and DayA) or 100 data points (synthetic scenarios). If each scenario led to the same outcome, similar distributions should be observed; however, Figure 5 illustrates divergent results that summarize the primary findings of this work.

Both typical day (DayA and DayD) distributions were associated with a much larger span and heavier tails than the other distributions, suggesting that contact dynamics variation between days was characterized by different distributions rather than the temporal variances in the disease model. Because daily contact density was not normally distributed, the means for the typical day scenarios are pulled higher by the long tails evident in Figure 5. Consistent with observations regarding the impact of aggregation in [25] and in entirely simulation-based studies, all the means (with the exception of SW2P) are greater than in the full detailed case, demonstrating the systemic overestimation introduced by aggregation of contact dynamics. Although the SW2P case outcome closely approximates the ground-truth outcome of FullD, it was the least accurate representation of the contact duration distribution among the small world representations. Increasingly accurate representations successively move the mean and overall distribution away from the FullD case (SW3P, SW3PTT), culminating in the empirically determined weight case SWE which had a mean coinciding with the FullA case – clearly demonstrating that seemingly more accurate attack rates (such as that associated with SW2P) can appear from a cancellation of offsetting biases.

### Impact of network structure

In line with previous simulation-centric studies [25], we found that aggregation has a tendency to overestimate the infection burden. As observed by [15], aggregation eliminates ordering information that would otherwise rule out possible transmission pathways among multiple individuals. Removing such constraints permitted simulated infections to spread more rapidly across the network. For example, if susceptible individual A contacts susceptible individual B earlier in a day and infective individual C (only) later in that day, C may infect A, but B will not be infected via A during that day. By contrast, aggregation over the course of a day would permit transmission not only from C to A, but also from C to A to B in that day.

It is common practice to calibrate models to historical data to raise confidence in a model’s predictive abilities [37,38]. Adjustment to edge connectivity or weights for agent-based simulations or to parameters such as the contact rate (*c*) for population-level simulations tune implicit assumptions regarding the underlying dynamic contact network. As the SW2P simulations demonstrate, principled estimates of contact strength or duration can lead to apparently excellent agreement with ground-truth scenarios; however, this was based on a flawed estimate of the actual contact data. Within this scenario, the highest contact duration 18 edges were trimmed from the contract graph as a result of the fit, leading to a systematic underestimate of the generally overestimated aggregate burden. For SW2P, two wrongs did in fact make a right: the reduced contact rate compensated for the implicit overestimate of infection transmission induced by network aggregation. The compensatory nature of such effects can be recognized by examining the effects of increasing fidelity with FullA in other synethic network scenarios. Systematically increasing the accuracy of the edge weight distribution, first by using a three-piece fit (SW3P), and then a 3 piece fit with included outliers (SW3PTT), increased the overall infection rate, until reaching the SWE scenario, whose mean corresponds closely with that of the FullA scenario.

### Impact of study period

**Figure 6:**
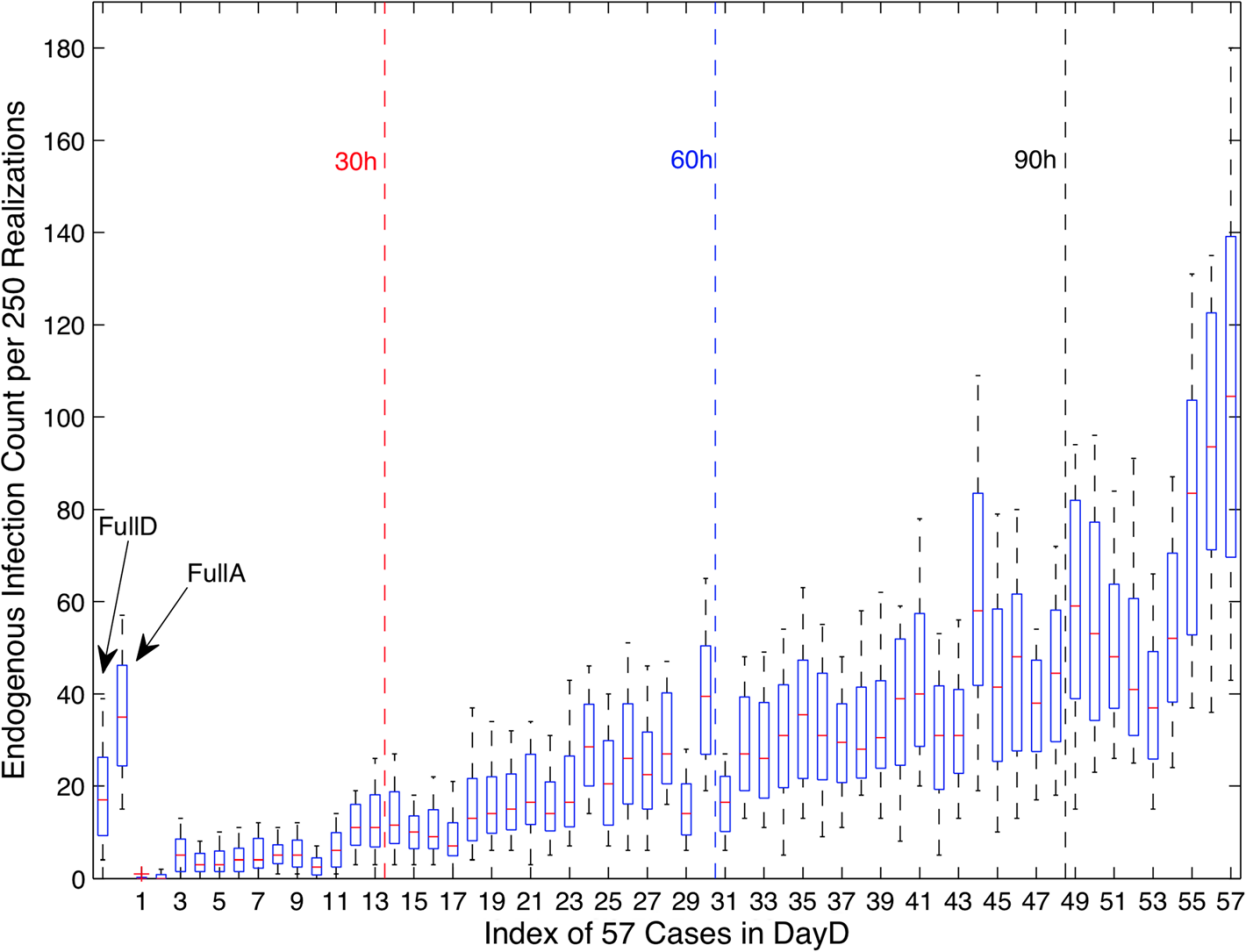
**Detailed Boxplot on Infected Count.** The number of endogenous infections for FullD, FullA and DayD, indexed by case pairing from 1–57 and sorted from lowest total daily contact time across all participants to largest total contact time. Four bins of contact time are denoted with dotted lines corresponding to less than 30 hours, between 30 and 60 hours, between 60 and 90 and greater than 90 hours of total contact time. Both mean and variance increase with total contact time, and match poorly with the mean and variance of the FullD case.

By collecting data on a hypothesized typical day [9], other researchers have in effect, exchanged study duration for study size; opting for shorter deployments but larger sample populations. The large variability of both aggregate and detailed day simulations in Figure 5, when compared with the various synthetic aggregate networks, indicates that the choice of day can have profound repercussions on the results of simulated infection transmission. To further investigate the scope of this variability, and to separate out the effects of model stochastics from those associated with choice of days, we subdivided each 10,000 realization ensemble into 40 ensembles of 250 realizations each. This step permitted the comparison between the distributions of the baseline (FullD and FullA) and each of the DayD scenarios, as shown in Figure 6. Each of the 57 cases in DayD are sorted and grouped by total contact duration.

While some stochastically induced variation is evident in the baseline scenarios (FullD, FullA), the variation between distributions when different days are chosen as typical and the variation among the realizations for many high contact duration days are substantially greater. The differences in endogenous infection counts between each day in the DayD scenario are easily accounted for when considering the probability of infection. On days with limited connectivity, there are both fewer transmission paths and fewer chances for an infectious event to result from a contact. As such, those few infectious events which do occur will tend to occur between few individuals, suppressing both the variance and the mean. In cases where many connections exist, the joint probability of infectious events and proximity increases, as do the number of individuals who could be infected. The impact of sampling error that can result from a typical day experimental design is apparent from the graph, but to more fully quantify this risk we attempted to determine how many of the 57 sampled day pairs could be viewed as typical.

**Figure 7:**
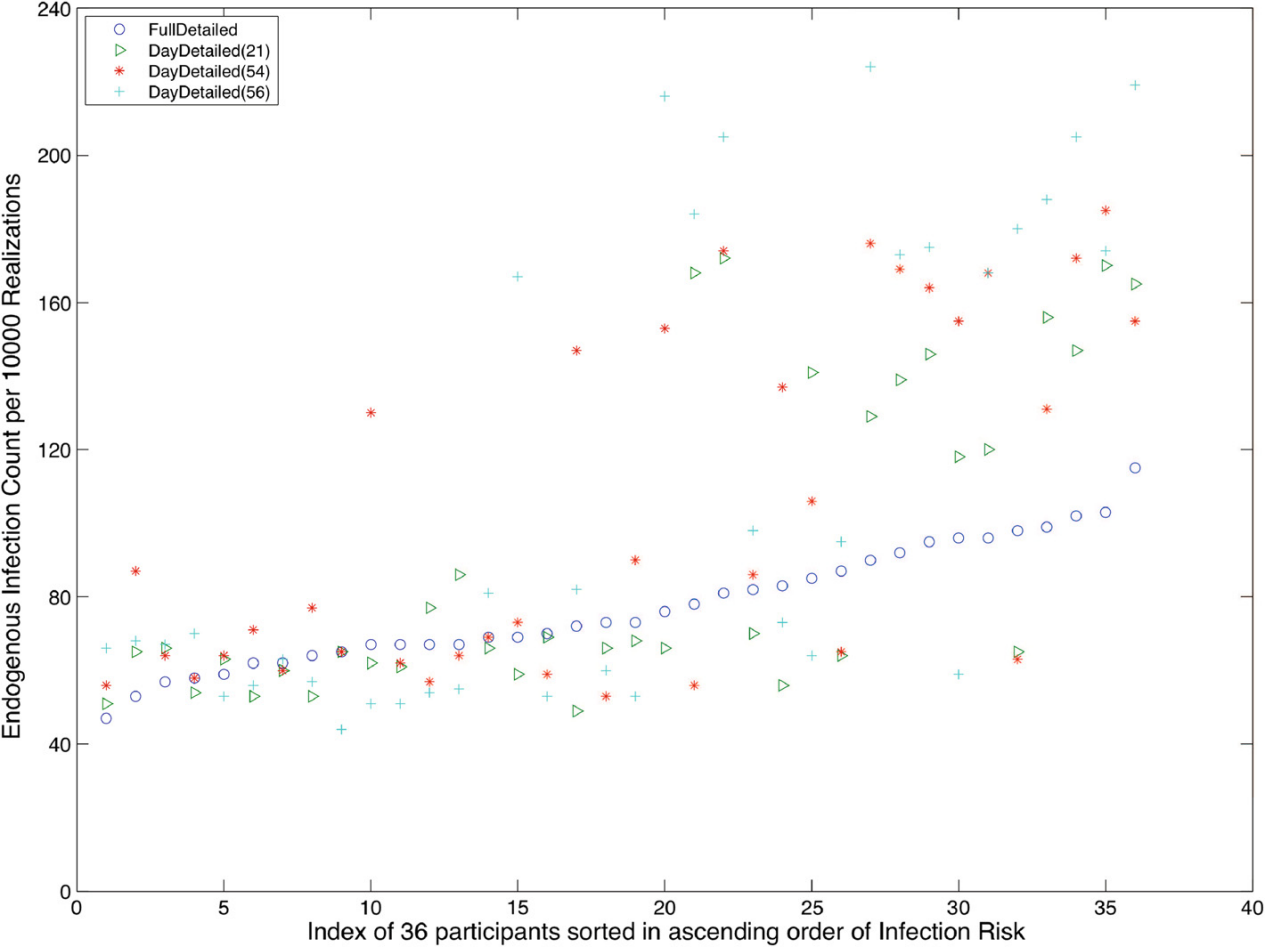
**Typical Day Infection Risk Per Person.** Infection risk for 36 participants of three selected cases of DayD networks that exhibit the highest infection rate correlation with the FullD scenario. The FullD risk (denoted by o’s) demonstrates a moderate increase in risk across participants. The days which correlate by infection count (+, *, ∆) show a marked difference between high-infection count and low infection count individuals.

We chose to define a typical day in a post-hoc fashion: typical days should lead to similar individual infection risks to the baseline network. To determine similarity, we computed Pearson’s correlation between each individual’s infection risk within the single case of the FullD scenario with that associated with each day-specific case in the DayD scenario. Only 3 day pairs (the 21^st^, 54^th^, and 56^th^) were found to have significant (ρ > 0.7, p < 0.05), correlation with the FullD scenarios, and no day pairs with strong and significant (ρ > 0.8, p < 0.05) correlations were found. Individual infection rates are shown in Figure 7, which clearly indicates the weakness of the correlation between the baseline FullD infection counts and each of the typical day realizations. Although reasonable matches exist for low-risk individuals (characterized by fewer infection counts in both the FullD and selected DayD cases), typical day techniques overestimated the infection risk for higher-risk individuals, leading to weaker correlations.

Based on our analysis, and subject to the limitation of our dataset, we concluded that typical day techniques can generate spurious rates of infection within closed populations and are unlikely to represent the richness of longitudinal datasets. Although we do not have the requisite study population to conclude that *T* (study duration) is more important than *N* (study sample size), Figure 7 indicates a much stronger variation between days than between people. Further evidence to this effect is provided by the coefficient of variation (*cv*) for infection rate, which has a value of 68.9 between participants and 80.6 between days.

## Discussion

The results presented here simultaneously underscore the high rate of return in terms of model reliability of investing in the collection of micro-contact data, but suggest that not all such investments confer equivalent value. It is widely recognized that model aggregation (for example, in the imposition of random mixing assumptions) tends to overestimate the spread of contagion. The results presented here suggest the natural extension of this understanding to the network context, where simulations employing purely static networks – such as might be produced by traditional network reconstruction techniques based on contact tracing, diarying or survey instruments – are also demonstrated to be biased towards overestimation of contagion. While this overestimation is pronounced in our experiments, it appears likely that the degree of this overestimation will depend heavily on the characteristics of the contagion process – particularly the speed of transmission relative to the speed of contact formation and dissolution [15]. The distortions associated with imposing a static network for some types of contagion suggest the importance of collecting information regarding network dynamics at a fine temporal granularity for such contexts. For very short-lived pathogens, a typical day approach may be sufficient in that all members of the network will be infected quickly or the infectious transmission will dwindle quickly. For long-lived pathogens such as tuberculosis, an aggregate model may be appropriate, as infection rates are low and latent periods are long. For the flu-like infection we modeled, our results indicate that typical days are too volatile and aggregate representations too permissive.

In addition to highlighting the importance of fine-scale data collection, these results also suggest a minimum efficient scale for such data collection. Such results are important in that many healthcare researchers have sought to defray the cost and logistic burden of electronically mediated micro-data collection by restricting the duration of studies. While such study designs sidestep many challenges associated with power consumption and device failures, they do rely heavily on the assumption that the network dynamics observed during the data collection interval is in some sense representative of the network dynamics over the long term. While such assumptions hold greater plausibility when applied in highly structured settings such as secondary schools [9], the results presented here highlight the elusiveness of identifying a typical day in some populations. Our results suggest that adopting a day as representative can be the source of major variability in simulation results, which can swamp the accuracy benefits conferred by using electronic micro-contact data. Diverse transmission modeling studies in the past several decades have revealed pronounced impacts of population heterogeneity on transmission dynamics, and have recognized the risks to model reliability of positing that the population is composed of homogenous typical persons. The results here suggest that – at least for some settings and sample populations – selecting a typical day can be fully as risky to model reliability as selecting a typical person – even when due diligence has been exercised to filter out manifestly unrepresentative days. While typical day assumptions appear to be hazardous in the context of our limited and highly clustered sample population, future data collection is required to determine whether similar risks extend from choosing typical days for studies with larger and more diverse populations.

Synthetic networks based on randomly generated networks and contact duration distributions are an appealing representation for extending results to larger populations. Our results highlight the need for care in the design of synthetic networks given that even high quality matches to the contact distribution using parametric distribution mixtures can lead to distortions of infection risks, primarily due to the impact of a few important outliers. When assessing the match fidelity between the synthetic results and results for the fully detailed data, it is easy to overlook an underestimation if it cancels out the overestimation due to an aggregated network whose outcomes misleadingly agree with historical or synthetic ground truth estimates. These biases could in turn distort the perceived trade-offs between interventions, or skew other important statistics regarding model dynamics. As is the case for random mixing models [4], even apparently calibrated models which match baseline data well can yield very misleading results when applied to counter-factual scenarios, such as interventions. We have little doubt that it is both important and feasible to build synthetic networks to capture transmission dynamics with high accuracy, but the current study suggests that achieving this goal will require moving beyond even well-calibrated models of network structure and contact duration. We particularly highlight the potential need to scrutinize the convenient assumption (applied here) of independence in the centrality of an agent and that agent’s contact duration distribution. While the close match in mean attack rates observed between the FullA and SWE scenarios suggests that this assumption may impose little distortion, further study is required to assess this assumption’s reliability.

The findings here have implications both for health research and epidemiological methodology. The results suggest that, while it is important to capture micro-contact data in many contexts, attempting to economize by reducing study duration may be penny wise and pound foolish. Use of micro-contact data appears to confer substantial benefits, but those benefits will be compromised – and potentially reversed – unless studies are of sufficient duration to capture day-to-day variability. While these results could be unique to smaller participant pools and flu-like infections, they likely apply to many important pathogens in high-risk populations of note such as institutional populations associated with long-term care facilities which are often not much larger than our participant pool.

We hope that the findings presented here will offer initial guidance in planning micro-contact studies, elevating the prospect that these studies will serve as a cost-effective means of enhancing health insight. Our results regarding the impact of network representation and parametric contact distribution approximations will also inform the data analysis methodologies used to analyze and generalize such data for transmission modeling. We have further described here a general methodology that can be replicated for future studies to assess the impact of aggregation on simulation results.

While our paper has made several important contributions to the field, as with all initial analyses, there are several shortcomings that should be addressed in future work. In fact, a major contribution of the paper is elucidating what the structure of future experiments should be to provide realistic samples of contact and disease dynamics for a given population. In particular, the role of sample size (*N*), versus study duration (*T*) is problematic.

Previous contributions [9,15] have opted for larger *N* at the expense of *T*, and attempted to use various techniques to extend the duration of simulations beyond *T* artificially. We have demonstrated that the error introduced through replicating *T* is significant and perhaps larger than using a well-tuned aggregate model. However, the limited participant pool of our study prevented us from addressing the issue of sample size. This leads to the possibility that our results are a statistical fluke, and that larger sample size would begin to diminish the heterogeneity in sample days to the point where one day was like any other for a large enough group of people. We believe this outcome to be unlikely due to the small-world nature of dynamic contact networks [36]. Rather than having an averaging effect, adding additional *N* – particularly for heterogeneous study populations – is likely to capture more partially isolated sub-networks with their own particular contact dynamic patterns and life rhythms. A study employing a typical day strategy must then assume that the sampling period represents a typical day for new subnets. In fact, we conjecture that larger sample sizes will increase the sensitivity to *T*, as temporal variability is likely to rise with population heterogeneity.

The uncertainty over the apparent tradeoff between study size and duration is a strong motivation for further contact dynamic studies with substantially larger, heterogeneous populations observed over longer time periods. Larger longitudinal studies would permit the analysis of the relative sensitivity of different disease models to simplifications in sample size, population and study duration by permitting knock-out experiments where similar simulations could be performed over statistically significant subsets of the data, and compared against each other and the full data set.

### Summary

We have demonstrated that the dynamics of empirical contact networks impact the outcome of simulation models, that aggregation over this data provides systematic overestimates of disease burden, that typical day data collection techniques employed elsewhere impose a risk. These findings are important as they inform both issues in microcontact study design and use of synthetic contact networks in calibrated models. However these findings are limited by the number of participants in the study and the single pathogen studied. In the future, we intend to investigate these impacts against larger, longer and more diverse datasets to determine if there is a point at which increasing study duration or sample size no longer materially affects simulation outcomes. We will also investigate the interaction between pathogen behavior and temporal sampling strategies. By providing this work we have established baseline insights into both the design of agent-based simulations of pathogen transmission and into the emerging discipline of contact dynamics acquisition and analysis.

## Competing interests

The authors declare that they have no competing interests.

## Authors’ contributions

MH designed and performed the simulations. WQ performed the synthetic network analysis. KGS and NDO conceived the study design and structure. All authors read and approved the final manuscript.

## Pre-publication history

The pre-publication history for this paper can be accessed here:

http://www.biomedcentral.com/1472-6947/12/132/prepub
